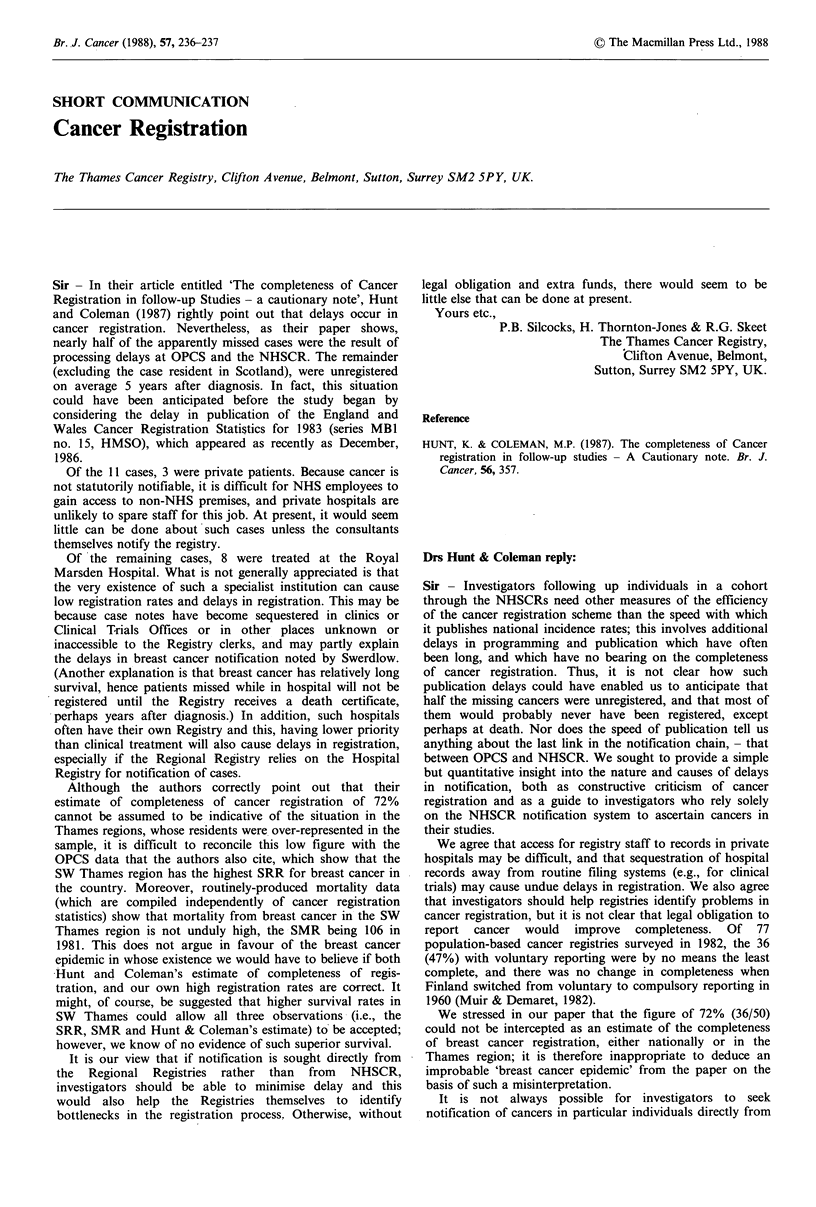# Cancer registration.

**DOI:** 10.1038/bjc.1988.50

**Published:** 1988-02

**Authors:** P. B. Silcocks, H. Thornton-Jones, R. G. Skeet


 © The Macmillan Press Ltd., 1988

SHORT COMMUNICATION

### Cancer Registration

*The Thames Cancer Registry, Clifton Avenue, Belmont, Sutton, Surrey SM2 5PY, UK.*

**Sir** – In their article entitled 'The completeness of Cancer Registration in follow-up Studies – a cautionary note', Hunt and Coleman (1987) rightly point out that delays occur in cancer registration. Nevertheless, as their paper shows, nearly half of the apparently missed cases were the result of processing delays at OPCS and the NHSCR. The remainder (excluding the case resident in Scotland), were unregistered on average 5 years after diagnosis. In fact, this situation could have been anticipated before the study began by considering the delay in publication of the England and Wales Cancer Registration Statistics for 1983 (series MB1 no. 15, HMSO), which appeared as recently as December, 1986.

Of the 11 cases, 3 were private patients. Because cancer is not statutorily notifiable, it is difficult for NHS employees to gain access to non-NHS premises, and private hospitals are unlikely to spare staff for this job. At present, it would seem little can be done about such cases unless the consultants themselves notify the registry.

Of the remaining cases, 8 were treated at the Royal Marsden Hospital. What is not generally appreciated is that the very existence of such a specialist institution can cause low registration rates and delays in registration. This may be because case notes have become sequestered in clinics or Clinical Trials Offices or in other places unknown or inaccessible to the Registry clerks, and may partly explain the delays in breast cancer notification noted by Swerdlow. (Another explanation is that breast cancer has relatively long survival, hence patients missed while in hospital will not be registered until the Registry receives a death certificate, perhaps years after diagnosis.) In addition, such hospitals often have their own Registry and this, having lower priority than clinical treatment will also cause delays in registration, especially if the Regional Registry relies on the Hospital Registry for notification of cases.

Although the authors correctly point out that their estimate of completeness of cancer registration of 72% cannot be assumed to be indicative of the situation in the Thames regions, whose residents were over-represented in the sample, it is difficult to reconcile this low figure with the OPCS data that the authors also cite, which show that the SW Thames region has the highest SRR for breast cancer in the country. Moreover, routinely-produced mortality data (which are compiled independently of cancer registration statistics) show that mortality from breast cancer in the SW Thames region is not unduly high, the SMR being 106 in 1981. This does not argue in favour of the breast cancer epidemic in whose existence we would have to believe if both Hunt and Coleman's estimate of completeness of registration, and our own high registration rates are correct. It might, of course, be suggested that higher survival rates in SW Thames could allow all three observations (i.e., the SRR, SMR and Hunt & Coleman's estimate) to be accepted; however, we know of no evidence of such superior survival.

It is our view that if notification is sought directly from the Regional Registries rather than from NHSCR, investigators should be able to minimise delay and this would also help the Registries themselves to identify bottlenecks in the registration process. Otherwise, without legal obligation and extra funds, there would seem to be little else that can be done at present.

Yours etc.,

P.B. Silcocks, H. Thornton-Jones & R.G. Skeet
The Thames Cancer Registry,
Clifton Avenue, Belmont,
Sutton, Surrey SM2 5PY, UK.

**Reference**

HUNT, K. & COLEMAN, M.P. (1987). The completeness of Cancer registration in follow-up studies – A Cautionary note. *Br. J. Cancer*, **56**, 357.